# Reframing climate change assessments around risk: recommendations for the US National Climate Assessment

**DOI:** 10.1088/1748-9326/aa7494

**Published:** 2017-07-21

**Authors:** C P Weaver, R H Moss, K L Ebi, P H Gleick, P C Stern, C Tebaldi, R S Wilson, J L Arvai

**Affiliations:** 1 Office of Research and Development, US Environmental Protection Agency, Research Triangle Park, NC, United States of America; 2 Joint Global Change Research Institute, Pacific Northwest National Laboratory, College Park, MD, United States of America; 3 Department of Global Health, University of Washington, Seattle, WA, United States of America; 4 Pacific Institute, Oakland, CA, United States of America; 5 Social and Environmental Research Institute, Northampton, MA, United States of America; 6 National Center for Atmospheric Research, Boulder, CO, United States of America; 7 School of Environment and Natural Resources, Ohio State University, Columbus, OH, United States of America; 8 School of Natural Resources and Environment, University of Michigan, Ann Arbor, MI, United States of America; 9 Author to whom any correspondence should be addressed.

**Keywords:** climate, assessments, risk framing

## Abstract

Climate change is a risk management challenge for society, with uncertain but potentially severe outcomes affecting natural and human systems, across generations. Managing climate-related risks will be more difficult without a base of knowledge and practice aimed at identifying and evaluating specific risks, and their likelihood and consequences, as well as potential actions to promote resilience in the face of these risks. We suggest three improvements to the process of conducting climate change assessments to better characterize risk and inform risk management actions.

Risk is a central concept in methods used to establish the objective significance of potentially damaging events (NRC 1983, Ruckelshaus 1983) or feelings associated with these threats to people and the things they value (Slovic *et al* 2004). It is widely accepted that risk is a function of three elements: the triggering event or hazard that could result in a potential loss, the magnitude of what stands to be lost, and judgments about the probability of the loss occurring—including the level of uncertainty bounding the probability estimate (NRC 1996). It is often the nature of the hazard and magnitude of the consequences that grab —or fail to grab—the attention of citizens and policy-makers, although, as we discuss below, other factors also play a role in how risks are perceived.

Climate change is increasingly being framed as a risk management problem, albeit a uniquely challenging one (King *et al* 2015, Sussman *et al* 2014). It is characterized by multiple intersecting and uncertain future hazards to natural and human systems, that are expected to unfold over a very large range of spatial and temporal scales, and whose probabilities may be difficult, or in some cases impossible, to quantify precisely (because of intrinsic and/or irreducible uncertainties about the future). It is a risk multiplier that interacts with other stressors to create new or alter existing risks (DoD 2014). The overall risk of severe outcomes stemming from climate change increases over time as greenhouse gas concentrations continue to rise, with the potential to affect multiple human generations across the full spectrum of cultural and geographic boundaries (IPCC 2014).

While effectively managing the long-term risks of climate change promises to be a difficult challenge, there are many risk-reduction actions that can be taken in the near term. We need, however, a knowledge base explicitly designed to support choosing and implementing the most appropriate and effective actions. Traditional climate change assessments are not optimally designed and constructed to deliver the kind of actionable information decision-makers need —and are asking for—to prioritize and manage climate change risks (Kirchhoff *et al* 2013, NAS 2016). From our collective experience as physical and social scientists working at the intersection of climate change and society, we argue it is time for a shift in the objectives and implementation of climate change assessments—from making what amounts to a general case for ‘action,’ to characterizing *specific* risks to help people develop, select, carry out, and monitor *specific* actions that ultimately have greater benefits than costs.

Society’s knowledge of current and future climate change has been established through decades of scientific research into the natural causes of past changes in climate, the buildup of anthropogenic greenhouse gases in the atmosphere, and the linked behavior of the oceans, atmosphere, ice, land, and biosphere. Social, political, and economic analyses of observed and projected climate impacts have provided further insights into how changes in the physical climate system can harm ecosystems, economic productivity, and human health and well-being. Climate change assessments are a key tool for organizing, summarizing, and communicating this science. An array of such assessments has been prepared under the auspices of international bodies such as the Intergovernmental Panel on Climate Change (IPCC), and in the United States through the US Global Change Research Program (USGCRP), established by a Presidential Initiative in 1989 and mandated by Congress in the Global Change Research Act (GCRA) of 1990^10^. These assessments have synthesized and communicated a robust understanding of the causes and proximate impacts of natural and anthropogenic climate change. They have built collective understanding within and across diverse scientific communities and focused attention on established findings and key areas for new research to address outstanding uncertainties.

An important stated goal of these assessments is to raise awareness among the public and policymakers of the existence, causes, and potential magnitude of human-induced climate change and its impacts. To a certain extent this has been successful: in the United States, for example, climate change is now recognized as a top risk to Federal government assets (GAO 2015), national security (DoD 2014, 2015), and business interests (Risky Business Project 2014, see also WEF 2016). Despite their technical sophistication and reach, however, major climate assessments have focused primarily on summarizing scientific understanding of, and scientific uncertainties about, expected changes in the physical climate system resulting from continued greenhouse gas emissions. Decision-makers, by contrast, need to understand how climate change may interfere with their plans and compromise their objectives, so they can adapt existing policies and adopt new strategies to stay on track—whether to protect life, health, and well-being, sustain economic growth, preserve natural resources, ensure continued performance of critical infrastructure, or maintain national security. The improved general understanding created by past assessments has led many decision-makers to demand that the next generation of assessments address new questions more relevant to their specific needs, which center on how climate change may intersect with their own unique decision imperatives; this embeds climate change within a larger environmental and socioeconomic context as one among many stressors (IPCC 2012, Weaver *et al* 2014, USGCRP 2016).

At the international level of the IPCC assessments, there have been increasing calls for changes in assessment focus, process, and structure, in recognition of this mismatch between demand and supply of information (Arvai *et al* 2006, Mach *et al* 2016, Hallegatte and Mach 2016, Kennel *et al* 2016). The need for change is arguably even more important for national assessments, which generally focus on climate change and impacts at national and sub-national scales, the scales of governance with primary decision-making authority for most response actions. One of the largest nationally-led assessments is the US National Climate Assessment (NCA), the most recent version of which, the Third NCA, was released in May 2014 (Melillo *et al* 2014). These NCAs are charged with integrating, evaluating, and interpreting the existing body of climate research, so as to assess current and projected trends in climate change and its impacts on US regions and sectors. Development of the Fourth NCA by the US government is underway, building on the success of, and lessons learned from, the Third NCA. Furthermore, the United States has recently committed to a long-term ‘sustained’ process of climate change assessment over multiple NCA cycles (USGCRP 2012, Buizer *et al* 2015), with a new Federal Advisory Committee dedicated to this sustained process^11^. Federal agencies, state and local governments, and businesses have begun preparing for adverse impacts of climate change on the basis of past assessments, but these actors need information more relevant to their planning and decision-making processes, which are fundamentally aimed at ensuring long-term resilience in the face of climate risks. Retooling US climate assessments around key societal risks would be a big step in that direction, and the time is right to do so. While our focus here is on national assessments, and in the United States in particular, the project of improving such assessments is an international one, with parallel efforts in other countries or regions (e.g. UK Committee on Climate Change 2017, California’s four Climate Change Assessments^12^); the need for peer learning between respective efforts is critical and often overlooked.

## Adapting core principles of risk assessment to climate:

To date, the approach of climate change assessments has primarily been rooted in communicating relative scientific certainty and uncertainty around anticipated changes in the physical climate system, along with some basic biophysical impacts that would seem to be generally implied by those climate changes: based, for example, on general understanding of associations such as those between impacts and weather extremes. Climate assessments have not typically characterized or analyzed specific societal risks resulting from climate change, including the socio-environmental determinants of vulnerability through which exposure to climate-related hazards creates risk.

The first step in that direction is to actually begin building technically sophisticated climate assessments around the core principles of risk assessment and risk management (see text box), which have been refined over many years across a diverse collection of disciplines (e.g. Kunreuther *et al* 2013, King *et al* 2015). Applying these principles to climate assessments will be challenging, in part because typical risk assessments focus on specific systems or situations, communicated to stakeholders who are directly affected by those risks. Climate change assessments, by contrast, tend to address a broad array of risks, many of them complex or indirect, relevant to many audiences with diverse concerns and exposures. Nevertheless, we argue that conscientious application of these principles can significantly advance climate assessment practice.

Drawing on these core principles, we suggest improvements in three areas of climate change assessment: (1) starting with a decision focus, (2) improving quantification of key risks relevant to users’ needs, and (3) presenting risk information strategically. Taken together, these improvements will lead to assessments that are more relevant and useful for decision-making.

## Starting with a decision focus:

We know from decades of research on judgment and decision-making that high-quality information alone will not necessarily lead to high-quality decisions (Fischhoff 2012). If supporting risk management decisions is to be the goal, then assessments must take as their starting point the needs of decision-makers by assessing risks in relation to their objectives. This includes identifying the values that may be at risk; synthesizing information on how climate risk management problems can be prioritized and manageably bounded; providing concrete options for managing risks, including how to create or identify such options; summarizing lessons learned in how to defensibly select among options by making explicit the inevitable tradeoffs that will arise when objectives conflict; evaluating the conditions under which such actions would be more or less effective; and providing guidance on how to manage continuous change over time, since climate risks are unlikely to be static. Climate assessments should identify the risks that are most salient for intended users, such as regional and sectoral stake-holders, recognizing that the assessment process itself may bring to the fore risks that users did not previously recognize as critical^13^. Importantly, these may include risks associated with potential risk management actions, which must be accounted for in evaluating the costs and benefits of action vs. inaction. No assessment can be all-encompassing in its treatment of climate-sensitive risks and decisions, but use of illustrative examples, case studies, and addressing classes of like decisions can provide real specificity (Kirchhoff *et al* 2013). Moreover, sequencing assessments such as the NCA over time can build a library of cases relevant to many decision-makers, across a range of plausible climate futures, geographic areas, and economic sectors, or in the face of emerging knowledge (NRC 2009).

None of this will be possible without engaging decision-makers and other affected parties from the very beginning of the assessment process, including at the scoping and question formulation phase, subsequently as authors, and finally in helping to convey assessment findings to diverse audiences. For climate change assessments to be effective in supporting risk management, they must be co-produced by subject matter experts and users (Lemos and Morehouse 2005, Meadow *et al* 2015, Moser and Davidson 2016). At a minimum, participation should be sought from key stakeholders and decision-makers; physical climate scientists, as well as natural and social scientists who understand the systems at risk; and experts in risk and decision sciences, public participation, and communication. It has long been recognized that two-way engagement between the producers and users of scientific information will not only make assessments more decision relevant, but also help to increase users’ understanding of and confidence in them (NRC 1996, Cash *et al* 2003). Linking risk characterization to potential response actions as a part of the assessment process, particularly in how assessment questions are framed in stakeholder engagement processes, may also lead to improved understanding of risks and options among assessment users (Gregory *et al* 2016). This type of deep, sustained engagement has rarely been put into practice at a large scale, however. Substantial initial efforts along these lines were pursued during the First NCA through stakeholder meetings and regional workshops (NAST 2001, Moser 2005), were revived during the Third NCA (Cloyd *et al* 2016), and should be expanded and institutionalized as a first step toward drawing stakeholders much more deeply into the assessment process.

## Improving quantification of key risks relevant to users’ needs:

The social construct of ‘value’ is at the core of risk assessment. Values are inherently subjective, come in many forms (economic, psychological, and otherwise), and can be challenging to quantify. Metrics of value pertain to life, well-being, property, economic prosperity, natural capital, ecosystem services, cultural heritage, and other qualities. Such valued attributes, to the extent they are vulnerable to climate change, should be the central focus of climate risk assessment. Our knowledge base, however, is insufficient to quantify the effects of climate change on many of these metrics because of conceptual challenges posed by climate change to standard assumptions, as well as practical data and methodological hurdles; insufficient understanding of how such metrics capture the way people actually feel; and, for non-monetized values, sometimes resistance to calculating them at all (Sussman *et al* 2014, Neumann and Strzepek 2014). Climate assessments thus usually ‘stop’ at enumeration of primary biophysical impacts (USGCRP 2011). Progress is urgently needed beyond this limited focus, with instructive examples in recent work (albeit primarily focused on economic valuation). For example, an analysis of the risks of sea level rise for the State of California evaluated the economic value of property at risk of flooding, as well as the size, economic status, and demographic backgrounds of the population living in areas vulnerable to flooding, area of wetland likely to be lost, and other metrics related to threatened transportation, energy, and water infrastructure (Heberger *et al* 2011). The impacts of harmful algal blooms (HABs) in the Great Lakes are being assessed using a range of economic metrics capturing the loss of services provided by the lakes (e.g. increased drinking water treatment costs, property value losses, beach closures), as well as the direct effects of toxic microcystin on public health (Bingham *et al* 2015, IJC 2013)—such events are expected to increase in frequency and severity in a changing climate (Michalak *et al* 2013). Finally, recent national-level assessments of the economic impacts of climate change in certain key sectors also provide a good foundation for next steps (Ciscar *et al* 2011, Houser *et al* 2015, EPA 2015, Burke *et al* 2015).

A second challenge for risk quantification in climate change assessments is to deal adequately with low-probability, high-consequence outcomes, which can dominate calculations of total risk, and are thus worthy of special attention. Without such efforts, we court the kinds of ‘failures of imagination’ that can prove so costly across risk domains (9/11 Commission 2004). Traditional climate assessments have focused primarily on areas where the science is mature and uncertainties well characterized (Kunreuther *et al* 2013). For example, in the IPCC or NCA lexicon, future outcomes are considered ‘unlikely’ if they lie outside the central 67% of the probability distribution. For many types of risk assessment (e.g. floodplain management, reinsurance, nuclear safety, air travel, toxicology), however, a 33% chance of occurrence would be very high; a 1% or 0.1% chance (or even lower probabilities) would be more typical thresholds (Bettis *et al* 2016, Hinkel *et al* 2015, Adams-Schoen *et al* 2015). The envelope of possibilities for which one must be prepared is often more important than the most likely future outcome, especially when the range of outcomes includes those that are particularly severe (de Perez *et al* 2014). Application of scientific rather than risk-based norms in communicating climate change uncertainty has also made it easier for policy-makers and other actors to downplay relevant future climate risks (Stern *et al* 2016, Ebi *et al* 2016). In fact, there is evidence that climate assessments have historically understated potential effects rather than overstated them (Socolow 2011, Brysse *et al* 2013).

To be useful in a risk context, climate change assessments therefore need a much more thorough exploration of the tails of the distributions of physical variables such as sea level rise, temperature, and precipitation, where our scientific knowledge base is less complete, and where sophisticated climate models are less helpful. We need greater attention on the strength of uncertain processes and feedbacks in the physical climate system (e.g. carbon cycle feedbacks, ice sheet dynamics) (NRC 2013), as well as on institutional and behavioral feedbacks associated with energy production and consumption, to determine scientifically plausible bounds on total warming and the overall behavior of the climate system (Heal and Millner 2014). Accomplishing this will require synthesizing multiple lines of scientific evidence, including simple and complex models, physical arguments, and paleoclimate data, as well as new modeling experiments to better explore the possibility of extreme scenarios. It will also require addressing the profound challenges these structural uncertainties pose to the traditional tools and methods of economic analysis (Stern 2013, Weitzman 2011).

Of course, none of this is to imply that science will be able to assign precise probabilities to such extreme outcomes in all or even most cases; with rare events, quantifying their likelihood is difficult. In this context, climate assessments could benefit from exploration of alternative uncertainty frameworks, such as possibilistic prediction (e.g. Betz 2010). Lack of complete knowledge need not delay decisions, however. Best practices in risk management tell us to be conservative in our assumptions and plans under such circumstances (Heal and Millner 2014), and assessments can convey where uncertainties mask large risks and where they do not. Assessments also need to separate uncertainties in knowledge of the climate system from uncertainties in knowledge of the vulnerability of social systems (Cutter and Finch 2008). For example, while there is lack of certainty about what could happen with land-falling hurricanes over this century, there is far too much evidence of the vulnerability of coastal regions to hurricanes and associated storm surges.

Extreme impacts can also result from complex interactions of physical events with biological, social, and economic systems, and these systems with each other, in a cascade of risk. The effects of climate change are likely to occur in an uneven manner, as there are nonlinearities in these systems and thresholds that may be crossed even with incremental shifts in the mean climate state (Ebi *et al* 2016, NRC 2012). For example, it was noted in the late 1990s that a reduction in average runoff in the Colorado River basin of 20% could lead to a drop in reservoir storage and hydropower generation by as much as 60%; similarly, the risks of large flood events in the basin increased in a highly non-linear fashion with modest increases in runoff (Gleick and Chalecki 1999). In many coastal cities, tidal flooding is recurring much more frequently today than only a few years ago, because of the small but inexorable year-by-year rise in sea levels, and the rate of recurrence is accelerating (Sweet and Park 2014). And considering the long lag between actions that commit the planet to the long-term impacts of climatic change, assessments also need to pay more attention to the potentially dramatic consequences of actions taken (or postponed) today. While systematically exploring these types of complex or systemic risks has not been a priority in past climate change assessments, it must become a central focus going forward.

## Presenting key risk information strategically:

Climate change assessments are not intended to serve as night-table reading; rather, they are intended to ‘collect and synthesize the rapidly evolving science and help supply timely and relevant information to decision-makers’ (Melillo *et al* 2014). Given the breadth of material to be synthesized, it is critical that the information provided by the assessment be strategically framed. The scientific community often shies away from the idea of framing, but the reality is that all assessments are framed, though often only implicitly. A frame communicates why an issue or decision matters, and is meant to simplify technical details and highlight which options or actions should be considered over others (Nisbet and Newman 2015). As a result, the way in which information in an assessment—and the assessment as a whole—is framed can have a significant impact on the degree to which risk-based information is useful for and useable in decision-making. Appropriate framing is thus a crucial part of effectively conveying risk-based information in climate change assessments.

Framing starts with a decision about *which* risks to present, highlighting the importance of beginning the assessment process with decision makers’ needs, as discussed above. This helps refine the quantification of risk by focusing on those pieces of information that will be most critical in specifically chosen decision contexts. Communication scholars have suggested that it may be the largest risks or costs that are most important when prioritizing information to include in an assessment meant to inform action (Nisbet and Newman 2015). In addition to choosing which risks, other insights from risk and science communication can make climate assessments more user-friendly by informing *how* to present the risks in a world constrained by time, material resources, and competing concerns (Nisbet and Mooney 2007). Best practice in risk communication offers insights ranging from how to present a quantitative evaluation of multiple risk management options, to more general insights about problem framing and presenting probabilities, consequences, and uncertainties in a format that is intuitively meaningful to diverse audiences (Morgan *et al* 2002). Best practices previously identified for the Third NCA included providing numeric (not just verbal) estimates of risk, using standardized likelihood ranges (e.g. very likely = > nine out of ten chance), and providing the 90% confidence range for findings. We should build significantly on these and related practices and continue to standardize their implementation.

In addition, there are other best practices that could be incorporated into future assessments^14^. For example, projected changes in temperature or precipitation and the likely impact on at-risk values may be better described using analogies to more familiar risks (though more research in evaluating the effectiveness of analogies as climate communication tools is needed; e.g. (Raimi *et al* 2017)). Presenting expected changes in absolute versus relative risk terms can be informative; a 50% reduction in the likelihood of a major drought is quite different when that reduction is from 100% to 50% versus 10% to 5%. Information on the uneven or inequitable exposure of vulnerable populations at risk may also help policy-makers prioritize responses. In addition, future assessments would benefit from better use of visual aids to demonstrate baseline or status quo risk versus the additional risk due to climate change or the reduced risk associated with action. Experience with characterizing non-climate disaster risks can also offer useful lessons (Glavovic and Smith 2014). Finally, assessments have relied almost exclusively on written reports to communicate their findings. The Third NCA experimented with alternative ways of communicating, including interactive and social-media-friendly web-based publication, short videos, and other types of communication materials and strategies (though there has been limited work to date to evaluate the additional reach resulting from these innovations). Finally, assessments should produce a broader range of technical content, including GIS tools, methods, and datasets from which customized maps, visualizations, and analyses can be developed to meet more specific user needs.

Despite the large body of scientific evidence on climate change, developed over decades, we have not generally been as successful in providing the kind of information decision-makers need to identify and prioritize the specific actions required to manage the many individual (and interconnected) risks of climate change. We need to do better, by turning significant attention toward building a robust practice of climate change risk assessment. The prescription we have put forward, for US national efforts but more broadly applicable, would be a good start.

## Text box on core principles of risk assessment

(adapted from King *et al* 2015)

- Define what we value (what is ‘at risk’); make this transparent and put these things (people, human systems, valued natural systems and services) front and center in the assessment. Risk analysis inevitably involves definitions of valued outcomes that reflect particular ethical or political interests. Open deliberation is required to define relevant values that are acceptable to all stakeholders (Fischhoff 2015).
- Define what we wish to avoid with respect to these valued things (e.g. thresholds of performance, viability, losses and damages). ‘What keeps you up at night?’ (Moser and Davidson 2016).
- Carry out analyses to identify what risky outcomes are possible—cannot be ruled out—starting with the biggest ones. In such analyses, it is useful to distinguish between two questions: ‘What is most likely to happen?’ and ‘How bad could things get?’ Other important questions include ‘What methods and tools are available to manage those risks?’ ‘What efforts are needed over short versus long timescales?’ and ‘By when are we likely to have additional information that may change our risk perceptions and/or decisions?’
- Distinguish between ‘direct’ risks, that deal with individual impacts resulting from a given change in climate, and might be the targets of adaptation actions at local and regional scales, and ‘systemic’ risks, that relate to major, potentially interconnected failures across multiple regions or sectors, and thus provide important motivation for adaptation and mitigation actions at larger scales (see also Kennel *et al* 2016).

## Footnotes

10 Public Law 101–606(11/16/90) 104 Stat. 3096–3104, signed into law by President George H.W. Bush.

11 www.federalregister.gov/documents/2016/03/31/2016-07208/united-states-global-change-research-program; one of the paper authors, RHM, serves as the Chair of this Federal Advisory Committee.

12 http://climatechange.ca.gov/climate_action_team/reports/climate_assessments.html.

13 See also literature contrasting ‘bottom-up’ and ‘top-down’ approaches to climate vulnerability and adaptation assessment (e.g. Wilby and Dessai 2010, Pielke *et al* 2012) and decision support (e.g. Weaver *et al* 2013).

14 See CRED (2014) for a summary of best practice in climate communication, NAS (2013) for a summary of best practice in science communication, and Fischhoff (2011) for a summary of best practice in risk communication.
